# The effect of speech–gesture asynchrony on the neural coupling of interlocutors in interpreter-mediated communication

**DOI:** 10.1093/scan/nsad027

**Published:** 2023-05-10

**Authors:** Xu Duan, Jie Zhang, Yi Zhang, Yuan Liang, Yingying Huang, Hao Yan

**Affiliations:** Key Laboratory for Artificial Intelligence and Cognitive Neuroscience of Language, Xi’an International Studies University, No. 6 Wenyuan South Road, Xi’an, Shannxi 710128, P.R. China; Department of Radiation Medicine, Air Force Military Medical University, No. 169 Changle West Road, Xi’an, Shannxi 710032, P.R. China; Key Laboratory for Artificial Intelligence and Cognitive Neuroscience of Language, Xi’an International Studies University, No. 6 Wenyuan South Road, Xi’an, Shannxi 710128, P.R. China; Key Laboratory for Artificial Intelligence and Cognitive Neuroscience of Language, Xi’an International Studies University, No. 6 Wenyuan South Road, Xi’an, Shannxi 710128, P.R. China; Key Laboratory for Artificial Intelligence and Cognitive Neuroscience of Language, Xi’an International Studies University, No. 6 Wenyuan South Road, Xi’an, Shannxi 710128, P.R. China; Key Laboratory for Artificial Intelligence and Cognitive Neuroscience of Language, Xi’an International Studies University, No. 6 Wenyuan South Road, Xi’an, Shannxi 710128, P.R. China; Department of Linguistics, Xidian University, No. 2 Taibai South Road, Xi’an, Shannxi 710071, P.R. China

**Keywords:** verbal communication, speech–gesture asynchrony, dialogue interpreting, neural coupling, fNIRS hyperscanning

## Abstract

In everyday face-to-face communication, speakers use speech to transfer information and rely on co-occurring nonverbal cues, such as hand and facial gestures. The integration of speech and gestures facilitates both language comprehension and the skill of the theory of mind. Consecutive dialogue interpreting (DI) allows dyads of different linguistic backgrounds to communicate with each other. The interpreter interprets after the interlocutor has finished a turn, so the interlocutor watches the gesture first and hears the target language a few seconds later, resulting in speech–gesture asynchrony. In this study, we used the functional near-infrared spectroscopy hyperscanning technique to investigate the influence of speech–gesture asynchrony on different levels of communication. Twenty groups were recruited for the DI experiments. The results showed that when the interpreter performed consecutive interpreting, the time-lagged neural coupling at the temporoparietal junction decreased compared to simultaneous interpreting. It suggests that speech–gesture asynchrony significantly weakened the ability of interlocutors to understand each other’s mental state, and the decreased neural coupling was significantly correlated with the interpreter’s interpretation skill. In addition, the time-aligned neural coupling at the left inferior frontal gyrus increased, which suggests that, as compensation, the interlocutor’s verbal working memory increases in line with the communication process.

## Introduction

During everyday communication, especially in face-to-face interaction, speakers use speech to convey information and rely on spontaneous gestures (McNeill *et al.*, 1994). These gestures include hand and facial gestures (Kendon, 2004; Bavelas *et al.*, 2014). Iconic hand gestures, in particular, visually represent information about object attributes, relations and actions (McNeill, 1992). Facial gestures include movements or positions of the eyebrows, mouth, head and so on (Kendon, 2004; Bavelas and Chovil, 2018). Gestures occur simultaneously with speech, and while speech is more important for conveying factual, abstract and persuasive information, gestures are more important for conveying messages related to impressions, relationships and affective states (Kendon, 1986; McNeill, 1992; McNeill *et al.*, 1994). Co-speech gestures and accompanying speech are thought to form an integrated system of meaning during language production (McNeill, 1992), called speech–gesture integration, through which the listener combines the information from both modalities into a single mental representation (Obermeier *et al.*, 2011).

Verbal communication requires the ability to generate and understand utterances, as well as infer the beliefs, desires and goals of others (Frith and Frith, 2006). The latter ability is also known as the theory of mind (ToM) (Premack and Woodruff, 1978). The abilities of comprehension and the abilities associated with the ToM have been shown to dissociate in individuals with aphasia (Apperly *et al.*, 2006; Willems *et al.*, 2011) and autism (Åsberg, 2010; Terzi *et al.*, 2016). A neuroimaging study has revealed that linguistic processing and ToM processing recruit distinct sets of brain regions (Jacoby *et al.*, 2016). Speech and gesture interact mutually and obligatorily in order to enhance language comprehension (Kelly *et al.*, 2010; Hostetter, 2011). Meanwhile, gestures, potentially aided by a mirror neuron system, may emphasize or aid in inferring the beliefs and intentions of others. Hand gestures were shown to evoke stronger activity in brain areas associated with social cognition, the ToM and personal knowledge (Montgomery *et al.*, 2007). Facial gestures, which represent the smallest nonverbal cues in recognizing emotions, can effectively convey the mental state of others (Baron-Cohen *et al.*, 1997; Ribeiro and Fearon, 2010).

Communication is usually carried out in the context of a shared language, but due to globalization, people of different cultural and linguistic backgrounds increasingly need to interact and communicate with each other (Nussbaum, 2014). Dialogue interpreting (DI) is defined as ‘interpreter-mediated communication in spontaneous face-to-face interaction’, involving at least two primary participants who do not understand each other’s language, and an interpreter who renders each participant’s message, utterance by utterance (Davitti, 2019). DI is typically conducted in consecutive mode, with the interpreter participating in the exchange during breaks by taking every turn (Bot, 2005). During turn-taking, listeners observe the speaker’s gestures and then hear the translated message a few seconds later. Similarly, the speaker makes an utterance and uses gestures, then waits a few seconds to hear the listener’s translated response. Thus, interpreter-mediated communication leads to a problem of speech–gesture asynchrony, in which speech and gesture are difficult to assemble (Habets *et al.*, 2011).

Recently, the hyperscanning technique (Montague *et al.*, 2002; Kelsen *et al.*, 2020; Cheng *et al.*, 2022) and the ‘two-person neuroscience’ perspective have been applied in studies of interpersonal communication and the human brain (Hasson and Frith, 2016; Redcay and Schilbach, 2019). These studies showed that language communication is associated with a pattern of neural coupling or interpersonal neural synchronization (INS) between partners (Garcia and Ibanez, 2014; Hirsch *et al.*, 2018), regardless of its physical structure (Honey *et al.*, 2012). Jiang et al. suggested that different levels of neurocognitive processes in interpersonal language communication are closely associated with distinctive patterns of INS (Jiang *et al.*, 2021). For example, neural coupling between interlocutors is in highly positive relationships with mutual understanding (Hasson and Frith, 2016), and an increase in INS was found when the teacher predicted the student’s knowledge state in a study of teacher–student hyperscanning (Zheng *et al.*, 2018). Functional near-infrared spectroscopy (fNIRS), since its introduction in the late 1970s (Jöbsis, 1977), has been increasingly applied in brain functionality research related to language communication (Dieler *et al.*, 2012; Wan *et al.*, 2018).

In this study, we investigated the influence of speech–gesture asynchrony on the neural coupling of cross-lingual interlocutors in interpret-mediated communication with respect to mutual understanding and the skill of the ToM. To this end, we designed two naturalistic DI conditions: the consecutive DI (time-delayed interpretation) and the simultaneous DI (time-aligned interpretation) (Sandrelli, 2017). We played the pre-recorded interpreted speech by the interpreter in consecutive DI to interlocutors simultaneously in simultaneous DI, pretending that the interpretation was performed by the interpreter in a booth because the interpreting quality of student interpreters in simultaneous interpreting (SI) was inferior to that of those in consecutive interpreting (CI). We calculated the spatially and temporally distinctive neural coupling of interlocutors by wavelet coherence (WTC) analysis on fNIRS data. Moreover, the time-delayed interpretation in DI would lead to a discontinuous output for the two parties, so we computed the time-lagged neural coupling to represent the temporal shift of both parties (Stephens *et al.*, 2010; Dai *et al.*, 2018).

## Materials and methods

### Participants

Sixty healthy students were recruited from Xi’an International Studies University. They were all native Chinese speakers, and English was their second language. Twenty student interpreters, with a mean age of 23.8 ± 1.3 years, were second-year graduate students majoring in interpreting. They had passed the China Accreditation Test for Translators and Interpreters (English Level 3 interpreting). Forty interlocutors were undergraduate students who were not majoring in interpreting and were required to have a score >70 in the Test for English Majors-Band 4 (TEM-4) and 85 in the Listening and Speaking Practice Test (English). Half of the interlocutors were randomly assigned to speak Chinese in the study, and the other half were assigned to speak English. No significant differences were found between the two groups with respect to age or TEM-4 scores (Chinese speakers: mean age = 21.75 ± 1.21, TEM-4 score = 78.9 ± 6.02; English speakers: mean age = 21.6 ± 1.46, TEM-4 score = 80.9 ± 5.87, *P*_age_ = 0.363, *P*_TEM-4_ = 0.147). Each three-member group consisted of a student interpreter and two interlocutors of the same sex to avoid the potential confounding effect of mixed-sex interactions. Three group members were not acquainted with each other prior to the experiment. All participants were right-handed with normal or corrected-to-normal vision and hearing. All participants provided written informed consent to participate in this study. The study protocol has been reviewed and approved by the Ethics Committee on Human Experimentation of the Key Laboratory for Artificial Intelligence and Cognitive Neuroscience of Language, Xi’an International Studies University.

### Experimental setup

**Fig. 1.**
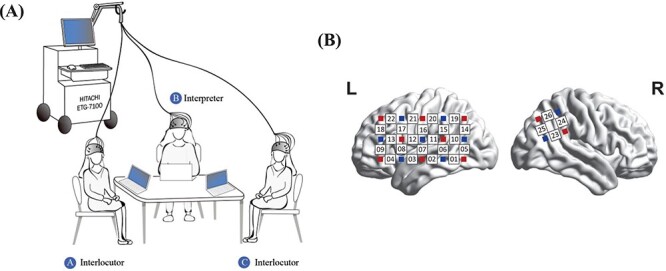
A schematic representation of the experimental design. (A) During the DI task, three participants sit around a table. Two interlocutors took turns speaking and listening in different languages, and the interpreter performed CI in both forward and reverse directions. (B) One 3 × 5 optode probe set was placed on the frontal, parietal and temporal lobes of the left hemisphere. Channel 3 was placed at T3 in accordance with the international 10–20 system. Another 2 × 2 optode set was placed on the rTPJ area, where the top right and bottom right optodes were placed at Cp4 and Cp6, respectively, following the international 10–10 system.

As shown in Figure 1A, to build a real-life scenario of DI, a pair of interlocutors sat on the opposite sides of a table and the interpreter sat between them forming an equilateral triangle. We defined the interlocutors who spoke Chinese or English as A or C, respectively, and the interpreter as B. Three members of the group wore optical caps to collect fNIRS signals simultaneously, and A and C wore earphones during the experiment. A 13-inch laptop was placed in front of each participant to display the experiment instructions and materials. Participants were allowed to interact face to face and were encouraged to express themselves through facial expressions and body gestures.

Groups completed three sessions over two sequential days. On the first day, they completed one session: cross-language communication under CI. On the second day, they completed two sessions in counterbalanced order: cross-language communication under SI and mother-tongue communication under the retelling of Chinese materials (hereinafter referred to as CC). Before the first session of the first day, they were asked to stay still, keep their eyes closed and listen to the sound of rain for 5 min. Before the first session of the second day, they were asked to stay still with their eyes closed for 5 min, but they did not listen to anything. The conversations and interpretations were audio-recorded throughout the experiments.

### Experimental procedures and materials

**Fig. 2.**
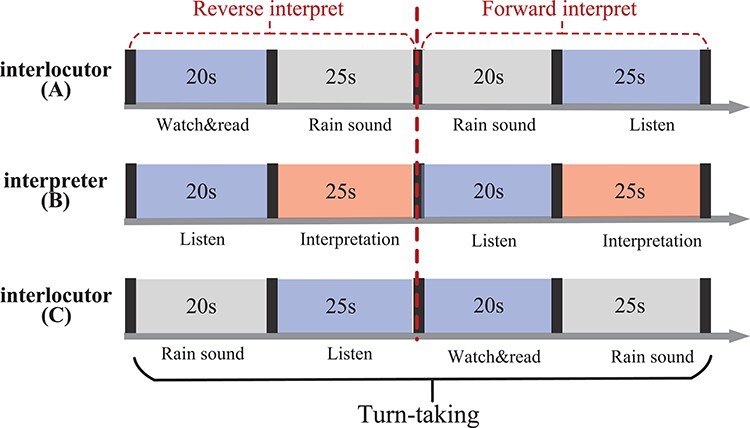
The experimental procedures for turn-taking. The first, second and third lines are procedures for interlocutor A, interpreter B and interlocutor C, respectively. In the first phase, interlocutor A saw Chinese material and read it aloud. Interpreter B listened and then interpreted the Chinese language into English, and interlocutor C listened to the English interpretation. In the second half of the turn, A spoke while C listened, and B performed forward interpretation.

Figure 2 shows the block structure in the CI session. The CI session was composed of two formal blocks, each consisting of five turn-takings. At the beginning of each block, a 30 s period was provided for the three participants to fix their gaze on a fixation cross-displayed on the screen to attain a steady state. During the first turn-taking, after a 2 s instruction, a Chinese paragraph was displayed on A’s screen for 20 s. The color of each word in the paragraph gradually changed uniformly, and A read the paragraph aloud to the interpreter at a rate matching the color changes. Meanwhile, the interpreter listened, prepared for interpreting and took notes if necessary. To prevent C from hearing their mother tongue, white noise in the form of rain sounds was played in C’s earphones. B then performed a reverse interpretation of Chinese to English for C within 25 s, at which A’s earphones began to play rain sounds to cover the English speech. The roles of speaker and listener were then swapped every 45 s. Next, C’s screen displayed an English paragraph, which C read aloud for 20 s at a color-changing rate while A listened to the rain sounds. B then performed a forward interpretation of Chinese to English within 25 s, with the rain sounds masking C’s speech. Following this, the second turn-taking commenced. The duration of each block was roughly 8 min, and the participants could rest at their discretion during the interval between blocks. The fNIRS caps were not removed or replaced between blocks on the same day, and prior to the formal block, the group had a chance to practice.

During the two sessions of SI, only two interlocutors sat on the opposite sides of a table wearing fNIRS caps and listening via earphones to the prepared interpretation recordings completed by interpreters the previous day. Each SI session consisted of two blocks, each containing eight turn-takings. In each turn-taking, A read aloud a color-changing Chinese paragraph displayed on the screen. C listened to the English interpretation from the earphones, which drowned out the Chinese language. Next, C read aloud an English paragraph, and the prepared English-to-Chinese interpretation was played simultaneously for A. Each block lasted ∼6 min. The experimental procedure was identical for the CC session and the SI session, except that the interlocutors heard the Chinese retelling, prepared in advance by the interpreter, in the CC session.

The conversation topics included the blockade in Coronavirus Disease 2019 (COVID-19) and exercise (CI), picnics and pets (SI) and Hong Kong tourism and movies (CC). The materials used for each topic were selected from the same level of teaching books, making their difficulty level equal. The materials did not contain technical, culture-specific or vague words.

### Assessment of comprehension and interpreting performance

When the group completed a formal block in three communication modes, we administered a behavioral assessment to the interlocutors to assess the amount of information transferred from the speakers to the listeners. The interlocutors had to answer three true or false questions related to the content of the conversation. The interlocutors must listen carefully and understand what the other person was saying so that they could answer each question correctly and rapidly. The total score for the three communication modes was 6 points.

Audio recordings of the interpretation in CI were submitted to five professional interpreter teachers for assessment. The interpreting performance in CI was scored separately for the Chinese-to-English and English-to-Chinese directions. Interpretations in Group 6 and Group 7 were not scored because the recordings from these two groups were lost. Assessments were made in three categories: content, form and delivery, with seven criteria in each category. The category score was given by completing the seven criteria, ranging from 0 to 10. The total score equaled the total weighted scores of three categories, with the weights of 2, 1 and 1, respectively (Lee, 2015). The assessment categories and criteria are listed in Supplementary Table S1.

### fNIRS data acquisition

fNIRS measurements were collected using an ETG-7100 optical topography system (Hitachi, Ltd, Tokyo, Japan). The optical density of near-infrared light at two wavelengths (695 and 830 nm) was measured, and the sampling rate was 10 Hz. We only employed oxygenated hemoglobin (HbO) concentration changes to estimate the neural coupling in this study, because changes in HbO hold the highest sensitivity to variations in the regional cerebral blood flow (Hoshi, 2007). Two optode probe sets, attached to a nylon cap, were positioned on participants’ scalps (Figure 1B). One $3 \times 5$ optode probe set (8 emitters, 7 detector probes and 22 measurement channels) was used to cover the left frontal, temporal and parietal lobes, which prioritize the brain’s language areas. The middle optode for the lowest probe row of the patch was placed at T3, following the international 10–20 system. Another $2 \times 2$ optode probe set (two emitters, two detector probes and four measurement channels) was placed on the right temporoparietal junction (rTPJ), where the top right and bottom right optodes were placed at Cp4 and Cp6, respectively, following the international 10–10 system. The distance between the emitters and detectors was 3 cm.

The optodes were accurately positioned before the first session of each group using a magnetic digitizer (PATRIOT; Polhemus, Vermont USA). The average Montreal Neurological Institute (MNI) coordinates and the associated brain regions of the 26 channels are listed in Supplementary Table S2. We conducted anatomical labeling of a spherical region centered on the projected point of each channel, with a default radius of 10 mm. We calculated the percentage (probability) of points belonging to each Brodmann area to the total spherical region (Tzourio-Mazoyer *et al.*, 2002). The Brodmann area with the highest percentage can be identified as the area covered by the channel.

### fNIRS data analysis

#### Preprocessing

The first and last 30s of the data in each task were removed to exclude the data when the fNIRS system was unstable. Functions in matlab toolbox NIRS-KIT (Hou *et al.*, 2021) were used to preprocess the data. First, detrending the raw fNIRS signal was done by subtracting the trend signal. Second, the temporal derivative distribution repair (TDDR) method was used to detect and correct motion artifacts (Fishburn *et al.*, 2019). Notably, filtering procedures were conducted after, rather than before, the calculation of neural synchronization (see *Determining the frequency ranges of interest*), because a full frequency range is suitable for calculating the INS in a study with a naturalistic design (Cui *et al.*, 2012; Jiang *et al.*, 2012).

#### Channel-wise INS

**Fig. 3.**
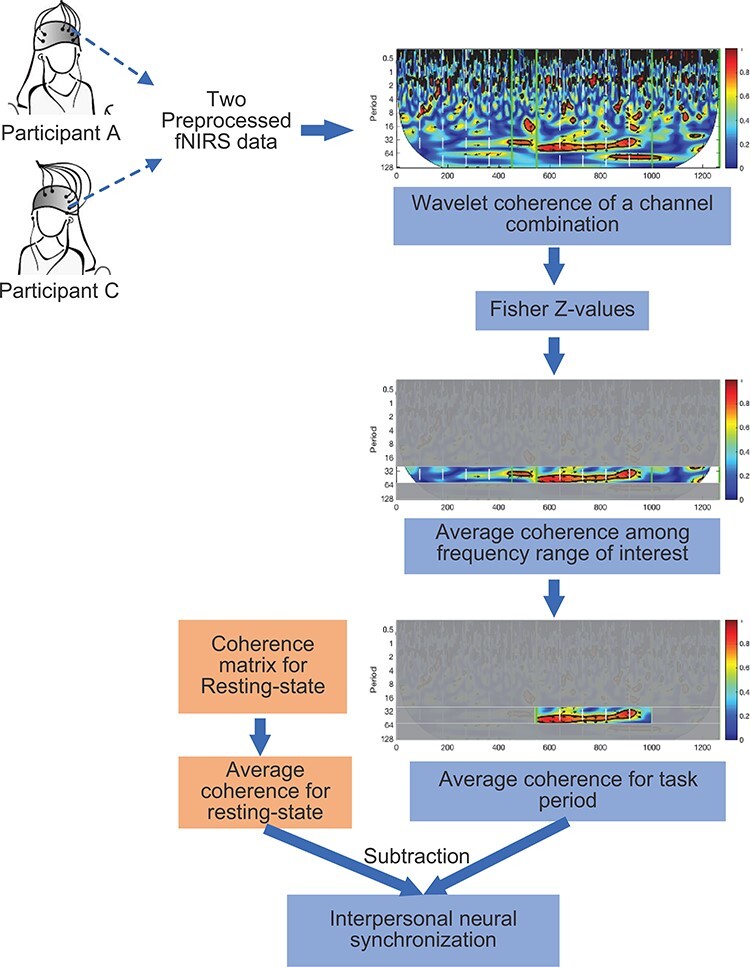
The flowchart for calculating channel-wise INS.

We utilized WTC to measure the INS between interlocutors (Cui *et al.*, 2012; Nozawa *et al.*, 2016; Liu *et al.*, 2019), taking into account the varying time scales of neural processes using localized wavelets (Torrence and Compo, 1998). The flowchart for calculating channel-wise INS is shown in Figure 3. We calculated WTC between two preprocessed fNIRS data from the two participants of a group for all possible channel combinations (i.e. $26 \times 26 = 676$ in total). The ‘wtc’ function with the Morlet wavelet in MATLAB was used to create WTC (Grinsted *et al.*, 2004). We set the minimum and the maximum scales as 0.3 and 128, respectively, with 106 frequency scales. A 2-D coherence matrix was obtained with specific frequencies and time points as columns and rows, respectively. We focused on the average coherence during the task period, which required the conversion of coherence values into Fisher’s *z*-values. For each communication mode and resting state with and without rain sounds, we examined the coherence matrix for all possible CH combinations between two participants and averaged them across the task period. We then subtracted the coherence value of the resting state from that of SI and CC, and the coherence value of the resting state with rain sounds from that of CI, resulting in the INS increase for CI, SI and CC (Cui *et al.*, 2012; Jiang *et al.*, 2012). Please find the MATLAB code on the website: https://github.com/duanxu007/Interpersonal-neural-synchrony-fNIRS-.

#### Determining the frequency ranges of interest

The frequency bands of interest were selected using a non-parametric multiple comparison correction method called the permutation test. This method identifies significant continuous clusters of interactions in the frequencies (Maris and Oostenveld, 2007; Zheng *et al.*, 2020; Long *et al.*, 2021). Specifically, we performed permutation sampling 1000 times under each condition and selected the *F*-values of the original sample that were higher than the first percentile of the permutation sample. The frequency ranges corresponding to these *F*-values were identified as the frequency ranges of interest.

#### Time lag in the INS between two communicators

To observe the lead-lag pattern of the INS increase, we calculated the coherence value by shifting the time course of C forward or backward relative to A by 1–26 s (step = 1 s). This involved delaying the fNIRS time series of C by −26 to +26 s compared to A, and calculating the INS increase of interlocutors using the delayed time series. This procedure was conducted for all tasks.

#### Group-level statistics

We averaged the INS increase in the frequency band of interest and conducted paired *t*-tests on the INS increase of all channel pairs in all time lags between CI and SI. The statistical results were subjected to Bonferroni multiple comparison correction for all channel pairs in all the time lags at *P* < 0.05 level. Subsequently, we performed a permutation test 1000 times in order to confirm the reliability of the INS.

#### Relationship between the interpretation score and the INS of interlocutors

Each turn-taking was split into two periods: the first 45 s with A speaking Chinese and C listening via reverse interpretation, and the next 45s with C speaking English and A listening via forward interpretation. After adjusting for the delay-to-peak effect in the fNIRS signals (∼6 s), we averaged the coherence values for each period of turn-taking. We then analyzed the relationship between the reverse interpretation score and INS increase in the first period, and the relationship between the forward interpretation score and INS increase in the second period separately.

## Results

### The frequency range of interest

**Fig. 4.**
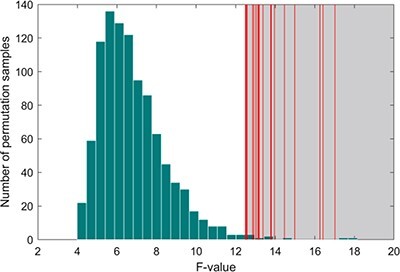
The results of frequencies of interest. The histogram represents the distribution of 1000 *F*-values for permutation samples, and the shadow denotes the area beyond the 99th percentile. Twenty verticle lines represent F-values corresponding to clusters of the original samples that fell into the shadow area.

The distribution of 1000 permutation samples is shown in Figure 4. The gray area beyond the 99th percentile indicates significance at the level of 0.01, with the red line representing the *F*-value corresponding to the cluster of the original sample that fell into the gray area. We calculated 20 frequency bands as frequencies of interest, with ranges that were relatively close. We combined these bands into a single set, which yielded two separate frequency bands: 31.5–50.0 s (0.020–0.032 Hz) and 12.5–19.8 s (0.050–0.080 Hz). The first period of the turn-taking was 45 s in CI, and 17–25 s was about the time for a single interlocutor to speak or listen. These bands included half of the turn-taking period and the full period of speaking or listening, indicating that the INS increases in these frequency bands were task-related.

### Effects of time-delayed interpretation on neural synchronization of interlocutors

**Fig. 5.**
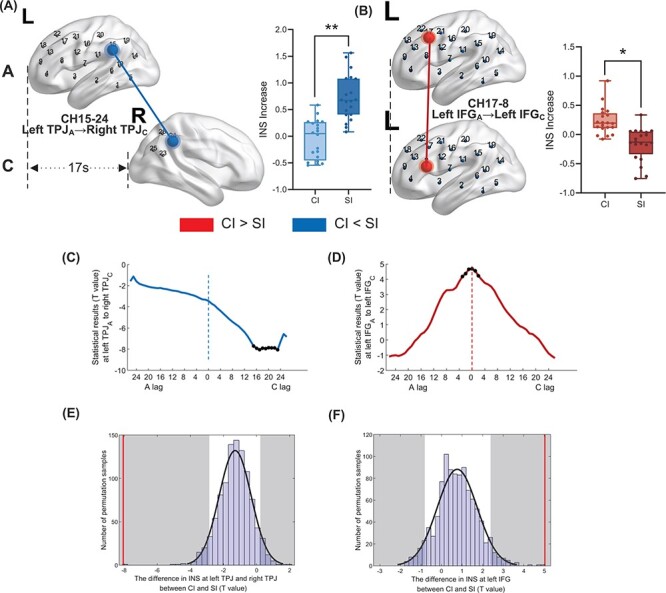
Neural synchronization differs between CI and SI. The results of *t*-tests revealed two significant channel pairs: (A) the left TPJ (temporoparietal junction) of A and the right TPJ of C (CI  < SI, frequencies 0.020–0.032 Hz, CH15–CH24) and (B) the left IFG (inferior frontal gyrus) of both interlocutors (SI < CI, frequencies 0.050–0.080 Hz, CH17–CH8). (C) and (D) The time-lagged effects. The significant *t*-values were observed across several time lags from C lag 17 s to C lag 23 s at left TPJ_A_ and right TPJ_C_, with the peak of C lagging behind 17 s of A, and from A lag behind 3 s to C lag behind 2 s at IFG_A_ and IFG_C_, with the peak of time-align. (E) and (F) The results of the permutation test. The histogram represented the distribution of the difference in the INS increase at left TPJ_A_ and right TPJ_C_ (E) and left IFG (F) for all permuted channel pairs. The *t*-values in the TPJ and the IFG of the original pairs were all significant within the 1% area (shadow area) after the Bonferonni correction. The *x*-axis represents the *t*-value, and the *y*-axis represents the number of permutation samples.

The differences in the INS increase between CI and SI were analyzed to investigate the effects of time-delayed interpretation on interlocutors. The *t*-tests on the INS increase between CI and SI showed that CI was significantly lower than SI at A’s left temporoparietal junction area (lTPJ) (CH15) and C’s right TPJ (rTPJ) (CH24), see Figure 5A, on the frequency range of 0.020–0.032 Hz, when C’s brain activity lagged behind that of A by 15–23 s, which reached a peak at a time lag of 17 s [*t*(2.38) = –8.054, *P* = 0.007;Figure 5C]. The red line fell into the left part of the gray shadow (two-tailed), indicating that the time-lagged INS increase at the lTPJ and rTPJ in the original pair was significant within the 5% area of the distribution of 1000 permutation pairs. With regard to the frequency range of 0.050–0.080 Hz, in contrast, CI was significantly higher than SI at A’s inferior frontal gyrus (IFG) (CH17) and C’s IFG (CH8) when A lagged 3 s to C lagged 2 s, which reached a peak at time-align (*t* = 5.060, *P* = 0.047) (Figure 5B and D). Figure 5F shows that the time-aligned INS increase at left IFG (red line) in the original pairs was significant within the 5% area (gray shadow) after Bonferroni correction.

To eliminate the impact of the INS increase when C was bilingual instead of a native English speaker, we also analyzed the differences in the INS increase of interlocutors between CC and SI. A series of *t*-tests on the INS increase of SI and CC showed no significant difference at any channel pairs in the frequency range of 0.050–0.080 Hz.

### The relationship between the INS increase of interlocutors and the interpretation score

**Fig. 6.**
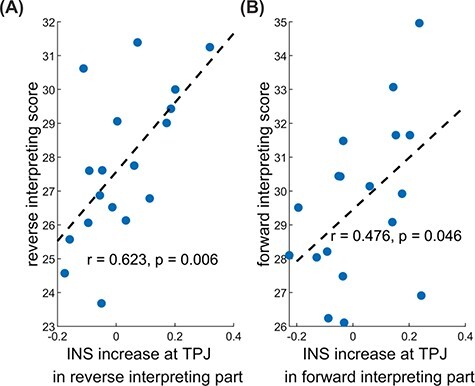
The relationship between the INS increase at the TPJ and the interpretation score. (A) In the A speaking and C listening period, the INS increase at the TPJ was significantly correlated with the reverse interpretation score. (B) In the C speaking and A listening period, the INS increase at the TPJ was significantly correlated with the forward interpretation score.

The Pearson correlations revealed that the average INS increase in the first phase significantly correlated with the reverse interpretation score (*r* = 0.623, *P* = 0.006) as shown in Figure 6A. In the second phase, the average INS increase at the TPJ significantly correlated with the forward interpretation score (*r* = 0.653, *P* = 0.003) as shown in Figure 6B. However, no significant correlations were found between the INS increase at the left IFG and the interpretation scores in the first or second phase.

### The results of the comprehension and interpretation scores

**Fig. 7.**
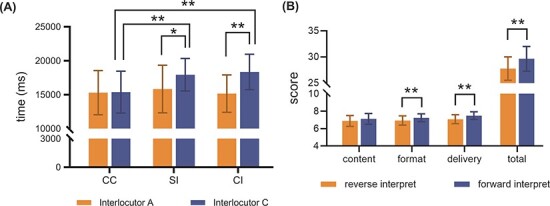
(A) The average answer time for A and C in three communication modes. (B) The average reverse and forward interpretation scores among 20 interpreters in content, format, delivery and total score. Significant differences are indicated as follows: *P < 0.05, **P < 0.01.

The average times for two interlocutors who communicated in CC, SI and CI are shown in Figure 7A. A $2 \times 3$ mixed analysis of variance (ANOVA) was used for the statistical comparison in terms of accuracy and time spent in answering, respectively. The statistical analysis yielded a significant interlocutor $ \times $ communication mode interaction [*F*(2.76) = 3.365, *P* = 0.040], partial (${\eta ^2} = 0.081)$. Post hoc comparisons revealed that C took significantly less time in CC mode compared to SI mode by 256 ms (*P* = 0.008) and compared to CI mode by 297 ms (*P* = 0.007). Additionally, a significant difference was found between A and C in both SI and CI modes, with A responding faster than C by 211  and 319 ms for SI and CI modes, respectively (*P* = 0.032 and *P* = 0.001). However, no significant results were found for content accuracy scores.

As shown in Figure 7B, in CI, the average interpretation score in the dimensions of content, format and delivery among 20 interpreters all fell within the extensive level (6.0–8.0). Kendall’s coefficients of concordance of the interpretation scores in three dimensions from five raters were 0.608, 0.672 and 0.581 (*P* < 0.001) in reverse interpretation and 0.579, 0.713 and 0.618 (*P* < 0.001) in forward interpreting, respectively, which suggested good reliability in each dimension. A *t*-test revealed significant differences between reverse and forward interpretations in form (*P* = 0.004), delivery (*P* = 0.003) and total scores (*P* < 0.001) and revealed no difference in content score.

## Discussion

When people with different backgrounds and languages need to communicate, DI provides a face-to-face method of helping people transmit knowledge across linguistic boundaries (Nussbaum, 2014). However, time delays in interpretation during DI lead to a problem of speech–gesture asynchrony for cross-lingual communicators. This study contributes to the literature on speech–gesture integration by manipulating the time delay in interpretation and investigating its effects on the neural synchronization of interlocutors. As represented by the hemodynamic signal of both interlocutors, the neural activity showed distinct synchronous patterns spatially and temporally during communication under time-delayed interpretation. On the one hand, when the interpreter performed CI, compared with SI, a significant decrease in INS was found when C’s brain activity in the rTPJ lagged behind that of A in the lTPJ by 17 s in 0.050–0.080 Hz, which was closely associated with the interpretation score. On the other hand, the change in time-aligned INS of interlocutors in the left IFG was significantly higher in CI compared to SI in the frequency range of 0.032–0.050 Hz and had no relationship with the interpretation score.

The bilateral TPJ is widely known as being associated with the ToM, the cognitive ability to predict others’ mental state based on various cues (Schurz *et al.*, 2017). Some researchers have found significant brain activity in the lTPJ and/or the rTPJ during ToM-related tasks by fMRI (Saxe and Wexler, 2005; Boccadoro *et al.*, 2019) and fNIRS (Bowman *et al.*, 2015; Zheng *et al.*, 2018). Moreover, the period of time lag (17s) roughly corresponded to the 20 s time delay of interpretation or the amount of time interlocutors waited for the transmission of the target language. Thus, the decreased INS with a time lag at the TPJ should reflect the function of the TPJ in predicting the mental state of the two interlocutors, and the interlocutors could only infer the mental state of the others by relying on the output of the target language. For connotative and emotional meanings or meta-messages about the state of an interpersonal state of mind, people depend largely on nonverbal signals, making nonverbal behavior especially important in interpersonal contexts (Burgoon *et al.*, 2011). When gesture and speech are out of sync, although the gesture is stored in memory in an abstract form, gesture cannot be integrated with the speech as the interlocutors wait longer. The interlocutors have to rely on the translated speech alone to infer the other’s state of mind and thus, resulted in a drop in the ToM. From another perspective, interlocutors in SI could make full use of speech–gesture integration, and they were better able to predict the mental state of others.

There was a relationship between time-lagged INS at the bilateral TPJ and the interpretation score, demonstrating that the change in the degree of speculation about each other’s intention, caused by delayed interpretation, is closely related to interpretation quality. That is, the higher the interpreter’s skill, the better interlocutors could understand each other’s intentions. It could be inferred that despite the influence of time-delayed interpretation, a skilled interpreter was still able to enhance the interlocutors’ ability to predict each other’s state of mind.

Notably, we may conclude that the time-delayed interpretation did not affect the mutual understanding of interlocutors, at least on the metric of behavior and neural synchronization. The ANOVA results showed no significant difference between CI and SI in terms of the accuracy and the time taken to answer. This lack of a significant difference was apparent whether interlocutors were acting as speakers or listeners, indicating a comparable level of mutual understanding. Stephens et al. found a strong positive correlation between the significant speaker–listener neural coupling at, for example, superior temporal gyrus/sulcus (STG/STS) and dorsal lateral prefrontal cortex (DLPFC) and the level of mutual understanding, that is, higher neural coupling between interlocutors indicated better mutual understanding (Stephens *et al.*, 2010). In fact, in our study, no significant difference in the INS was found between CI and SI at STG or DLPFC. In sum, in the case of cross-language communication, time-delayed interpretation negatively affected the extent to which each interlocutor could predict the other person’s mental state, though the two interlocutors could achieve a mutual understanding of the cross-linguistic content.

Another significantly increased synchronous neural pattern was the time-aligned INS in the left IFG. Although the IFG was traditionally thought to be associated with speech production, the left IFG, including BA44 and BA45, has been shown to be involved in verbal working memory (VWM) (Rama *et al.*, 2001; Zhu *et al.*, 2020). VWM refers to the temporary maintenance and manipulation of linguistic information in memory. The left IFG was regarded as the executive center for VWM, where verbal information storage and updating converge (Zhang *et al.*, 2003). Moreover, in one study, the activation of the IFG and the posterior parietal area was positively correlated with working memory load when prolonging the memoranda $n$ that is manipulated in active storage during an $n$-back task (Honey *et al.*, 2000). Zhu et al. also demonstrated that the anodal stimulation to the left IFG improved the response efficiency of an auditory 3-back task (Zhu *et al.*, 2020).

Thus, the increasing INS in the left IFG revealed a more heavily loaded VWM. One possible explanation is that the time spent waiting for the interpreter to transmit information to the other person brought about a strain on the interlocutor’s VWM, as they needed to remember what they said 20 s ago. Another explanation could be that when the gesture is not in synchrony with speech, more controlled, active memory processes are necessary to combine the gesture fragment and speech context (Habets *et al.*, 2011; Obermeier *et al.*, 2011). In addition, the VWM load caused by time-delayed interpretation persisted during the communication process and did not change with the interpreter’s skill level. We may regard the increased time-aligned INS at the left IFG as a constant compensation consequence.

In contrast to most research on neural synchrony, we employ it as a tool to investigate asynchrony in verbal communication. In addition to its empirical contribution, we believe it carries a certain theoretical merit. Our findings demonstrated a dissociation between comprehension and ToM abilities by using technical neural synchronization. When individuals are unable to integrate nonverbal cues and speech, the ability to comprehend speech may be unaffected, but the ToM ability suffers. Our results may provide relevant theoretical implications for the understanding of communication disorders such as autism spectrum disorders (ASDs) (Piazza *et al.*, 2021). Children with ASD rarely use social cues to shift their attention to other people’s facial expressions or eye gaze, leading to poor ToM and empathy skills (Baron-Cohen *et al.*, 1985; Harmsen, 2019).

A limitation of the experiment is that the C participants were Chinese bilinguals instead of native English speakers. It has been difficult to recruit native English speakers in our country recently due to the COVID-19 pandemic. However, the study findings revealed that replacing native English speakers with Chinese bilinguals had no impact on the INS of cross-language communication. There was no significant difference observed in the INS increase at any channel pair when comparing Participant C communicating in their mother tongue and in their second language. The statistical analysis of accuracy and response time revealed that the Chinese bilinguals comprehended the information equally well in both languages. However, it took them longer to comprehend the second language.

## Conclusion

In conclusion, we used an fNIRS hyperscanning approach to investigate the influence of speech–gesture asynchrony on different levels of interpreter-mediated communication. The results showed that the speech–gesture asynchrony led to a decreased time-lagged neural coupling at the bilateral TPJ area, which is closely associated with the interpreter’s skill level, as well as an increased time-aligned neural coupling at the left IFG area. The results indicated that the speech–gesture asynchrony negatively affected the extent to which each interlocutor could predict the other person’s mental state because he or she could only rely on the translated speech alone. A skilled interpreter could enhance the interlocutors’ ability of the ToM. Meanwhile, the load of VWM increased as a constant compensation during communication. We may suggest that the primary speaker take shorter turns in DI to obtain a better communication quality.

## Supplementary Material

nsad027_Supp

## Data Availability

The data underlying this article will be shared on reasonable request to the corresponding author.
