# Supplementary material for: The effect of speech–gesture asynchrony on the neural coupling of interlocutors in interpreter-mediated communication
Source: Soc Cogn Affect Neurosci. 2023 May 10;18(1):nsad027. doi: 10.1093/scan/nsad027 (PMC10243907; doi:10.1093/scan/nsad027)
Supplement: nsad027_Supp [file nsad027_supp.zip › scan-23-003-File009.docx]

**Supplementary Table 1**: Assessment categories and criteria for the interpretation scoring

| Assessment categories | CONTENT | | FORM | | | DELIVERY | |
| --- | --- | --- | --- | --- | --- | --- | --- |
| Criteria | ① No opposite meanings  ② Accurate rendition of main ideas  ③ No unjustified change in meaning  ④ Logical cohesion  ⑤ High level of completeness of information (except numbers and names)  ⑥ Accurate rendition of numbers and names  ⑦ No unjustified additions | | ① No incomplete sentences  ② Natural/idiomatic target-language expressions  ③ Unambiguous and clear diction  ④ Appropriate register and speech level  ⑤ Little source-language interference  ⑥ Correct terminology  ⑦ Grammatical correctness | | | ① Fluency of delivery (general concept/ impression)  ② No significant repairs or backtracking  ③ Impression of confidence  ④ Few fillers, hesitations and pauses  ⑤ Lively intonation and stress  ⑥ Finishing interpretation within the time limit  ⑦ No slips of the tongue | |
| Points | 10.0 - 8.0 | 8.0 - 6.0 | | 6.0 - 4.0 | 4.0 - 2.0 | | 2.0 - 0.0 |
| Levels of effectiveness | Complete | Extensive | | Moderate | Limited | | Zero |
| Characteristics | ALL characteristics present | MOST characteristics present | | SOME characteristics present | FEW characteristics present | | NO characteristics present |

**Supplementary Table 2**: The mean 3D MNI coordinates and the associated brain regions of the 26 channels

| Channels | Hemisphere | x | y | z | Brodmann Area | Probability |
| --- | --- | --- | --- | --- | --- | --- |
| CH01 | Left | -67 | -48 | -15 | 20 - Inferior Temporal gyrus | 0.600 |
| CH02 | Left | -71 | -21 | -9 | 21 - Middle Temporal gyrus | 0.947 |
| CH03 | Left | -61 | 6 | -4 | 48 - Retrosubicular area | 0.410 |
| CH04 | Left | -53 | 42 | 0 | 45 - pars triangularis Broca's area | 0.613 |
| CH05 | Left | -62 | -64 | -5 | 37 - Fusiform gyrus | 1.000 |
| CH06 | Left | -70 | -40 | 3 | 22 - Superior Temporal Gyrus | 0.533 |
| CH07 | Left | -67 | -7 | 13 | 22 - Superior Temporal Gyrus | 0.441 |
| CH08 | Left | -59 | 22 | 16 | 45 - pars triangularis Broca's area | 0.513 |
| CH09 | Left | -47 | 49 | 15 | 46 - Dorsolateral prefrontal cortex | 0.607 |
| CH10 | Left | -68 | -50 | 11 | 22 - Superior Temporal Gyrus | 0.476 |
| CH11 | Left | -69 | -23 | 24 | 2 - Primary Somatosensory Cortex | 0.495 |
| CH12 | Left | -64 | 3 | 27 | 6 - Pre-Motor and Supplementary Motor Cortex | 0.503 |
| CH13 | Left | -49 | 38 | 27 | 45 - pars triangularis Broca's area | 0.963 |
| CH14 | Left | -58 | -69 | 22 | 39 - Angular gyrus, part of Wernicke's area | 0.769 |
| CH15 | Left | -67 | -41 | 31 | 40 - Supramarginal gyrus part of Wernicke's area | 0.535 |
| CH16 | Left | -64 | -9 | 37 | 43 - Subcentral area | 0.460 |
| CH17 | Left | -52 | 19 | 40 | 44 - pars opercularis, part of Broca's area | 0.741 |
| CH18 | Left | -37 | 46 | 35 | 46 - Dorsolateral prefrontal cortex | 0.676 |
| CH19 | Left | -59 | -58 | 41 | 39 - Angular gyrus, part of Wernicke's area | 0.675 |
| CH20 | Left | -62 | -26 | 48 | 3 - Primary Somatosensory Cortex | 0.292 |
| CH21 | Left | -54 | 4 | 46 | 6 - Pre-Motor and Supplementary Motor Cortex | 0.870 |
| CH22 | Left | -39 | 29 | 49 | 9 - Dorsolateral prefrontal cortex | 0.930 |
| CH23 | Right | 64 | -59 | 21 | 22 - Superior Temporal Gyrus | 0.443 |
| CH24 | Right | 65 | -47 | 38 | 40 - Supramarginal gyrus part of Wernicke's area | 0.882 |
| CH25 | Right | 56 | -71 | 27 | 39 - Angular gyrus, part of Wernicke's area | 1.000 |
| CH26 | Right | 56 | -60 | 48 | 39 - Angular gyrus, part of Wernicke's area | 0.657 |
